# Collecting resource use data for economic evaluation in a prison setting with a focus on self-harm: the Prison Data Inventory (Self-Harm) (PDI (SH))

**DOI:** 10.3389/fpsyt.2025.1648044

**Published:** 2025-11-20

**Authors:** Louise Robinson, Kerry Gutridge, Rachel Meacock, Barbara Barrett, Kathryn M. Abel

**Affiliations:** 1 Division of Psychology and Mental Health, University of Manchester, Manchester, United Kingdom; 2 Lancashire and South Cumbria NHS Foundation Trust, Manchester, United Kingdom; 3 Division of Population Health, Health Services Research and Primary Care, University of Manchester, Manchester, United Kingdom; 4 Institute of Psychiatry, Psychology & Neuroscience, King’s College, London, United Kingdom; 5 Greater Manchester Mental Health Trust, Manchester, United Kingdom

**Keywords:** self-harm, prison, cost, resources, cost-effectiveness

## Abstract

**Introduction:**

Self-harm is a common and escalating problem in prisons, particularly in women. Understanding and measuring the costs of self-harm allow high-quality evaluation of new interventions. Here, we describe the co-development of a new resource inventory, the Prison Data Inventory (Self-harm).

**Methods:**

An existing forensic resource use tool, the SF-SUS, was adapted with prison staff and people with experience of being in prison.

**Results:**

Piloting showed that the tool takes researchers approximately two hours to complete per person.

**Discussion:**

This tool will allow use of existing prison data to improve the evidence base for reducing self-harm in prisons. In turn this will enable policy makers and commissioners to allocate resources most effectively, improving clinical outcomes.

## 1 Introduction

Self-harm rates in prison are high and rising, particularly among women. In England and Wales in the 12 months to March 2025 the rates of self-harm, numbers of self-harm incidents and the proportion of the prison population who self-harmed were the highest ever recorded for both men and women (1). However, the rate among women (5906 incidents per thousand prisoners) was more than eight times higher that among men (684 incidents per thousand prisoners); the number of incidents per self-harming woman was also higher than for men (16.4 versus 4.5); and the proportion of women self-harming was 326 per thousand in contrast with 152 per thousand men (1).

The average cost of hospital care for a self-harm episode in one UK general hospital has been estimated at £809 (2). A study of a UK general hospital cohort showed that patients with a history of self-harm had significant variation in their cost to health and social services, with self-harm repetition associated with increased resource use (3). Self-harm also incurs personal costs for prisoners in terms of physical injury, death and distress. Prisoners who do not self-harm are also adversely affected (4), as are staff (5, 6). For the NHS and prison service, self-harm in prison generates economic costs, including prison officer supervision, medical and psychological treatment and hospital transfer in severe cases. Understanding these economic costs is essential to evaluate the cost-effectiveness of interventions aimed at preventing or treating self-harm.

Despite the significant burden of self-harm, there are ‘remarkably few’ economic evaluations of interventions for self-harm and suicide (7). Most rely on secondary data and, while the majority suggest that interventions are cost-effective or cost saving, results from RCTs are mixed. Crucially, none have been conducted in prisons, taking account of unique costs such as prison officer time, escorts, and the Assessment Care in Custody and Teamwork (ACCT) process. The WORSHIP (Women’s Self-Harm in Prison) studies have developed an intervention to prevent self-harm that is tailored to prisons (8). However, a lack of pre-existing information on prison-specific resources relating to self-harm for either men or women has made a detailed cost-effectiveness assessment of the intervention challenging (8).

Comprehensive economic evaluation requires detailed description of resources used in the absence of intervention, as well as the identification and measurement of relevant costs and outcomes of the intervention (9). This allows accurate value-for-money estimates and enables resources to be moved or allocated more effectively. In prison, obtaining these data can be challenging. Access to prisoners is restricted, and while records of services and contacts are generally kept on site, they are often incomplete or difficult to access. Individual-level rather than average service use and, therefore, cost data are important for economic analysis to allows costs to be linked to individual outcomes. This is particularly important for self-harm which is extremely heterogeneous; some individuals may harm themselves frequently but without the need for medical intervention, while others may harm themselves less often but much more severely. A service-use questionnaire that recognises this heterogeneity, while remaining practical and accessible in prison settings, is key for accurate economic evaluation.

This paper describes the co-development, with prison stakeholders, of the Prison Data Inventory (Self-harm) (PDI(SH) – a tool designed specifically for capturing individual-level self-harm related resource use in prisons.

## 2 Methods

**Figure 1:**
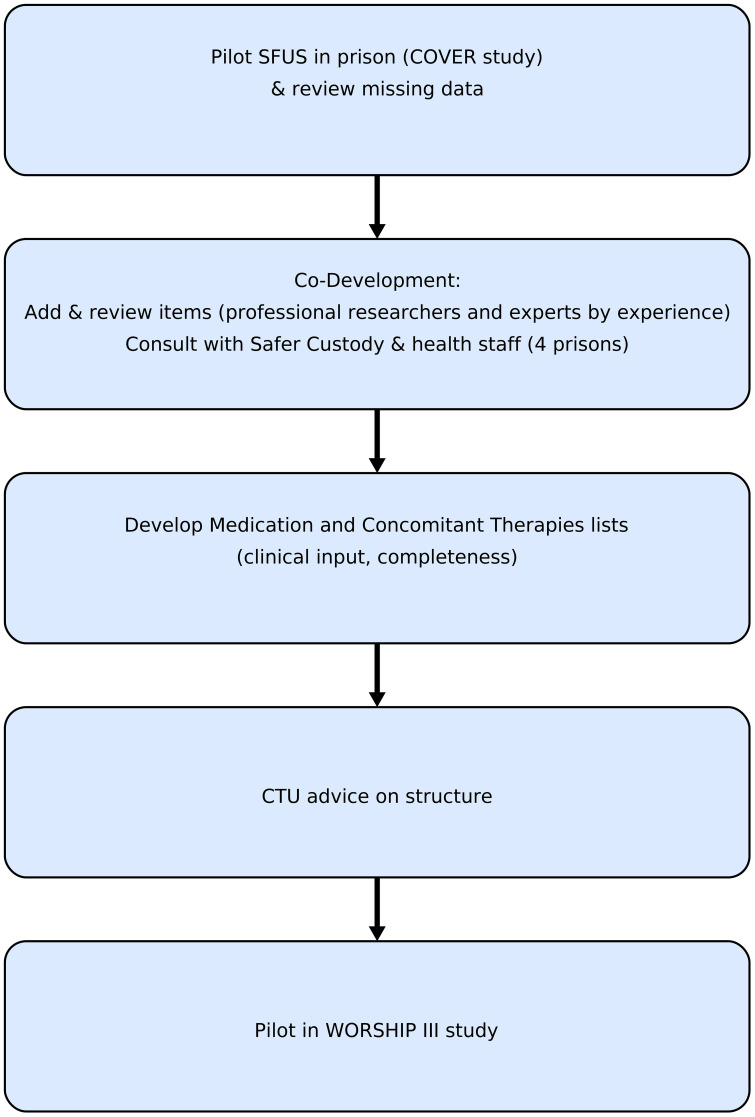
Iterative method of tool development.

We used a consultative approach to co-develop the tool with researchers, experts by experience, prison and prison healthcare staff during two prison studies of interventions for women who self-harm. After each stage, the tool was reviewed and revised with the team health economist (RM) (**Figure 1**). Ethical approval for the work was covered by the ethical approvals for these studies.

We first tested the Secure Service Use Schedule (SF-SUS) (10), an existing tool designed to retrospectively capture individuals’ use of services in prisons or secure psychiatric facilities, during a prison-based study of medical skin camouflage for women who self-harm in prison (the COVER study) (11). This tool was piloted to collect individual-level resource-use data for the women in the study. Information was obtained from two electronic systems used in prisons: SystmOne, the electronic health record; and the National Offender Management Information System (NOMIS), the operational database used to manage offenders. We encountered difficulty collecting information on external services, daily activities and court appearances and in verifying absence or missingness in data. Often, missing information was recorded elsewhere in the prison or was not routinely recorded at all. We concluded that an adapted version of the SFSUS, co-created with prison staff, would be more effective.

We worked with the team health economist (RM) to identify the initial limitations of the SF-SUS. We examined which resource-use categories had missing data and explored why, whether the information was missing at source, not recorded or recorded elsewhere.

### 2.1 Co-development consultations

The COVER research team – comprising prison researchers with expertise in self-harm and clinicians with experience managing self-harm in prison- added categories relevant to self-harm and its management. We reviewed the revised tool with two members of the team who were experts-by-experience (women with lived experience of self-harm in women’s prisons) to ensure all relevant self-harm related items were included.

We then conducted detailed consultation interviews with at least one member of Safer Custody and one of healthcare staff at four closed women’s prisons in England involved in the WORSHIP III study (https://fundingawards.nihr.ac.uk/award/16/111/51). These consultations focused on the availability of resource-use data, its location within prison systems, and procedures for access.

Clinical members of the research team reviewed the sections relating to medication and psychotherapeutic groups to ensure that appropriate categories were included. Once the prototype tool and appendices had been developed, the King’s Clinical Trials Unit gave advice on the structuring of responses to avoid free-text entries. The revised tool was then piloted during the WORSHIP III study.

## 3 Results

### 3.1 Revisions to the SF-SUS

#### 3.1.1 Review of use in COVER study

The COVER study review, including input from experts-by-experience, resulted in several revisions. These included adding prison-specific self-harm items that incur use of prison resources (**Table 1**). For example, the ACCT (Assessment, Care in Custody and Teamwork) process, used to assess and manage risk of self-harm in prison and led by prison Safer Custody departments, requires risk assessments, multidisciplinary meetings, planned ‘scheduled’ conversations and observations. All off these activities incur staff time, an opportunity cost. Other self-harm-specific items were also added, such as distraction packs, alternative clothing, and physical treatment of injuries such as cleaning materials or sutures. Use of segregation was adapted to include Rule 45 (segregation for an individual’s own protection). Categories that were part of the original SF-SUS, but not available in prison systems, for example court appearances, were removed (**Table 2**).

**Table 1:** Additional items added to the tool.

ACCT: reviews, scheduled conversations
Resources for self-harm used e.g. distraction packs
Alternative Clothing
Physical Treatment of Self-Harm
Segregation to include use of the Rule 45 segregation order

**Table 2:** Items removed from the tool.

Accommodation
Daily Activities (other than therapeutic groups- moved to separate log)
Court Appearances
Tribunals/Security Category Reviews

#### 3.1.2 Treatment review

The clinical review of the medication section found that participants in the COVER study (11) were prescribed high numbers of medicines, with some individuals receiving more than 100 different prescription items during their 12 weeks in that study. To make data collection more efficient and relevant, the team agreed that the tool would focus on psychotropic and pain medication, which relate most directly to mental health, injuries, or chronic pain. An intervention relating to self-harm was considered unlikely to affect physical health conditions significantly. Clinically-trained researchers devised comprehensive list of medications for mental health conditions, substance misuse or pain. This ‘concomitant medications’ list also required that the researcher specify, if possible, the indication for the prescription e.g. pain relief, epilepsy, psychiatric indication as these drugs have several potential indications. The list is provided in **Appendix A**.

In the SF-SUS, information on therapeutic groups is entered as free text. In prisons, a wide variety of therapies and talking treatments are provided by different providers, e.g. prison healthcare, in-reach mental health teams, Safer Custody, education departments and third sector organisations. These therapies include ‘mental health’ interventions e.g. for anxiety or substance misuse, ‘forensic’ interventions targeting offending behaviour, and interventions targeting other issues such as parenting. Therapies may be given individually or in groups, and while some use one treatment modality e.g. CBT, others will combine modalities in one treatment. Several therapies offered were aimed at more than one problem, for example personality disorder and literacy, and were informed by more than one approach, such as psychoeducation and CBT. We categorised therapies into six groups: individual or group psychotherapy; delivered by healthcare, prison or third-sector providers. For each intervention, the name and a description were recorded.

In COVER, it was difficult to obtain a comprehensive list of all therapies an individual was undertaking from prison or health databases, as record keeping is divided among providers. To address this in the PDI(SH), we decided that the most accurate method of collecting information on concomitant therapies was to interview participants about which groups, courses or treatments they were attending. Missing information should then be checked with the provider. The clinical review of the tool also identified which therapies could relate to self-harm outcomes.

#### 3.1.3 Health economist revisions

The health economist (RM) incorporated the use of ACCT and physical treatments for self-harm injuries to ensure these could be included in economic evaluations. Hospital outpatient attendance was separated from hospital admissions. Items on the SF-SUS which would not incur a cost to the prisons or NHS and therefore could not be calculated, were removed (**Table 2**).

Definitions of missing data were clarified as ‘Researcher unable to access source’ and ‘Search not completed by researcher, needs search’, to improve the distinction between data absence and incomplete searches.

#### 3.1.4 Prison staff consultation

Consultation with Safer Custody and healthcare staff at four closed women’s prisons established whether the data corresponding to all items in the tool could be accessed in each prison, the location of the information in prison, and how it could be accessed. Safer custody staff provided prison-specific information about the ACCT process and prison segregation. While healthcare data was largely available on SystmOne, the average length of appointments was not routinely recorded. This was ascertained this through consultation with healthcare staff, including mental health in-reach teams.

### 3.2 The Prison Data Inventory (Self-harm) (PDI(SH))

The resulting PDI(SH) is an eight-item tool which captures resource use across the following categories:

1. ACCT (contacts, dates opened and closed).
2. Items Given to Help with Self-Harm (e.g. distraction packs).
3. Alternative clothing provision.
4. Professional contacts with healthcare providers.
5. Physical treatment for self-harm injuries.
6. Healthcare admission within prison.
7. Overnight stays in hospital outside prison, including required escorts.
8. Outpatient/emergency department visit from prison, including required escorts.
9. Segregation.

The concomitant medications appendix records psychotropic, pain and substance use-related medication prescriptions, date of initiation/termination, dose and frequency, and indication. The therapies appendix records therapy name and date, aims, delivering organisation, delivery mode (individual or group), number of sessions attended per week, and end date if relevant.

The final PDI(SH) consists of a researcher-completed questionnaire which has two appendices (therapy; medication). A short interview with the participant is recommended where possible to verify current therapy information as these are often delivered by multiple providers and can be difficult to identify solely from records.

### 3.3 Piloting

Piloting with three women across two prisons in the WORSHIP III study showed that completing the PDI(SH) and appendices takes approximately two hours per participant. Completion involves the researcher collecting resources from data sources including NOMIS, SystmOne, the ACCT database (where one is held), individual ACCT paper folders, Safer Custody logs and segregation unit folders.

## 4 Discussion

Rates of self-harm in prison, particularly in women, remain alarmingly high (12) and there is an urgent need for effective and cost-effective interventions. Our research with women and men in prison (https://fundingawards.nihr.ac.uk/award/NIHR201504) has identified that a number of different approaches have been implemented in prisons without evidence of their cost-effectiveness. Published evaluations of self-harm or suicide interventions have not included cost-effectiveness analyses (13–15). High-quality, reliable and prison-specific resource use data are therefore essential to inform development and implementation of interventions. These data are not straightforward to collect and previously no tailormade tool was available with which to do so. The PDI (SH) addresses this gap by enabling systematic collection of comprehensive, individual-level data for women in prison, using primary sources. The tool captures costs not only relating to self-harm but broader service use, making it suitable for use in studies of other interventions in prisons.

### 4.1 Strengths and limitations

A major strength of the PDI(SH) is its co-production. The tool was developed collaboratively with researchers, prison staff, healthcare professionals and women with lived experience of prison. This approach ensured that it captures relevant resource categories for cost evaluation, but it also includes practical knowledge about where data are located and how they can be accessed.

Another strength is that the PDI(SH) does not rely on self-report, reducing the burden on participants in prison and instead uses existing data sources to gather information, increasing objectivity. However, data collection in prisons remains challenging. Barriers to access, particularly security restrictions, and limited accuracy of primary records, may limit reliability. Additionally, broader resources provided by other sectors with no direct cost to prisons or NHS, such as the Listener scheme, were not included. This means that costs such as the time or emotional distress of other prisoners are not captured by this tool.

The SFUS, on which this tool is based, was developed for the UK Dangerous and Severe Personality Disorder programme, focused on men’s special units in prisons and hospitals. While self-harm is a problem in both women’s and men’s prisons, processes for managing data may differ between the women’s and men’s estate. This tool would benefit from further piloting in men’s prisons to ensure that their data systems are captured before being used in studies with men.

Under ethics, policy and law, prisoners are entitled to the same quality of healthcare as people in the community (16–18). However, the cost of self-harm in prisons has not been formally evaluated. Addressing this is urgently needed given the high prevalence and impact of self-harm.

## 5 Conclusions

The PDI(SH) allows the collection of detailed, prison-specific, individual-level service use data for the first time, to allow economic understanding of self-harm and evaluation of interventions. It captures both prison and NHS costs, and its use will help policymakers allocate limited resources more effectively, ensuring that interventions implemented in prison are cost-effective. We recommend its use in future evaluations of self-harm interventions in prison.

## Data Availability

The original contributions presented in the study are included in the article/**Supplementary Material**. Further inquiries can be directed to the corresponding author.
