# Supplementary material for: Collecting resource use data for economic evaluation in a prison setting with a focus on self-harm: the Prison Data Inventory (Self-Harm) (PDI (SH))
Source: Front Psychiatry. 2025 Nov 20;16:1648044. doi: 10.3389/fpsyt.2025.1648044 (PMC12676598; doi:10.3389/fpsyt.2025.1648044)
Supplement: Supplementary file 1 [file Table1.docx]

**Concomitant Medications List**

| **1. Acamprosate calcium**  **2. Agomelatine**  **3. Alprazolam**  **4. Amisulpride**  **5. Amitriptyline**  **6. Aripiprazole**  **7. Armodafinil**  **8. Asenapine**  **9. Atomoxetine**  **10. Benperidol**  **11. Bupropion**  **12. Buspirone**  **13. Carbamazepine**  **14. Chlordiazepoxide**  **15. Chlorpromazine**  **16. Citalopram**  **17. Clobazam**  **18. Clomipramine**  **19. Clonazepam**  **20. Clorazepate**  **21. Clozapine**  **22. Desvenlafaxine**  **23. Dexamfetamine**  **24.Diamorphine hydrochloride**  **25. Diazepam**  **26. Disulfiram**  **27. Donepezil hydrochloride**  **28. Dosulepin**  **29. Doxepin**  **30. Duloxetine** | **31. Escitalopram**  **32. Fluoxetine maleate**  **33. Flupenthixol**  **34. Flupentixol**  **35. Fluphenazine Decanoate**  **36. Flurazepam**  **37. Fluvoxamine**  **38. Gabapentin**  **39. Galantamine**  **40. Guanfacine**  **41. Haloperidol**  **42. Hydroxyzine Hydrochloride**  **43. Imipramine**  **44. Isocarboxazid**  **45. Lamotrigine**  **46. Levetiracetam**  **47. Levomepromazine**  **48. Lisdexamfetamine mesilate**  **49. Lithium carbonate**  **50. Lithium citrate** | **51. Lofepramine**  **52. Lofexidine hydrochloride**  **53. Loprazolam**  **54. Lorazepam**  **55. Lormetazepam**  **56. Lurasidone**  **57. Melatonin**  **58. Memantine Hydrochloride**  **59. Methadone hydrochloride**  **60. Methylphenidate**  **61. Mianserin**  **62. Midazolam**  **63. Mirtazapine**  **64. Moclobemide**  **65. Modafinil**  **66. Nalmefene**  **67. Naltrexone hydrochloride**  **68. Nitrazepam**  **69. Nortriptyline**  **70. Olanzapine**  **71. Omega 3 acid ethyl esters**  **72. Oxazepam**  **73. Paliperidone**  **74. Parenteral B12**  **75. Paroxetine** | **76. Pericyazine**  **77. Perphenazine**  **78. Phenelzine**  **79. Phenytoin**  **80. Pimozide**  **81. Pipotiazine**  **82. Pramiprexole**  **83. Prazosin**  **84. Pregabalin**  **85. Promazine**  **86. Promethazine hydrochloride**  **87. Propranolol**  **88. Quetiapine**  **89. Reboxetine**  **90. Risperidone**  **91. Rivastigmine**  **92. Sertraline**  **93. Sulpiride**  **94. Temazepam**  **95. Topiramate**  **96. Tranylcypromine**  **97. Trazodone**  **98. Trifluoperazine**  **99. Trimipramine**  **100. Trio-iodothyronine**  **101. Tryptophan**  **102. Valproate/valproic acid**  **103. Venlafaxine**  **104. Vortioxetine**  **105. Ziprasidone**  **106. Zolpidem tartrate**  **107. Zopiclone**  **108. Zuclopenthixol. 109. Other**  **0. No concomitant medications reported during the trial** |
| --- | --- | --- | --- |
