# Supplementary material for: Collecting resource use data for economic evaluation in a prison setting with a focus on self-harm: the Prison Data Inventory (Self-Harm) (PDI (SH))
Source: Front Psychiatry. 2025 Nov 20;16:1648044. doi: 10.3389/fpsyt.2025.1648044 (PMC12676598; doi:10.3389/fpsyt.2025.1648044)
Supplement: Supplementary file 2 [file Table2.docx]

**Prison Data Inventory – Self-Harm (PDI-SH)**

Participant ID Number: _____________________________________

Time period data collection:

- From: ___ ___/___ ___/___ ___ ___ ___
- To: ___ ___/___ ___/___ ___ ___ ___

Completed by: ______________________________________

Completion date: ___ ___/___ ___/___ ___ ___ ___

**Instructions**

The form is completed from prison databases. Information on psychotherapeutic interventions must be supplemented by participant interview.

- Confirm data sources with Safer Custody and Healthcare
- If data may be duplicated across sources, check all.
- Use accurate dates for independent verification.

**Missing Data Codes**

| **Situation** | **Code** | **Action** |
| --- | --- | --- |
| Source inaccessible | **888** | Notify Trial Manager |
| No search | **999** | Notify Trial Manager |
| Source checked no record | **0** | Use only if verified |

**1. ACCT Records**

**ACCT Periods**

Where can I find this information? NOMIS, ACCT database

| **Date ACCT Opened** | **Date ACCT Closed** |
| --- | --- |
|  |  |
|  |  |
|  |  |
|  |  |
|  |  |
|  |  |
|  |  |
|  |  |
|  |  |
|  |  |
|  |  |
|  |  |
|  |  |
|  |  |
|  |  |
|  |  |
|  |  |
|  |  |

**Need more space?** Attach a continuation sheet with participant ID and date

**ACCT Meetings and Reviews**

For each ACCT period, record**:**

1. Initial assessment interview - date, duration and staff occupations
2. Case reviews – date, duration and occupation of all staff
3. Additional one to one meetings with Safer Custody staff

Where can I find this information?

Individual ACCT folders (also called diaries/books), case reviews also on NOMIS

**Staff Occupation Codes**

| **Role** | **Code** | **Role** | **Code** |
| --- | --- | --- | --- |
| Prison Custody Officer | 1 | GP | 10 |
| Senior Custody Officer (ACCT Mgr) | 2 | Substance Misuse Staff | 11 |
| Safer Custody Officer | 3 | OMU Caseworker | 12 |
| Deputy Governor/Senior Manager | 4 | Chaplain | 13 |
| Key Worker/Personal Officer | 5 | Education | 14 |
| Mental Health Nurse | 6 |  |  |
| Other Nurse | 7 |  |  |
| Psychiatrist | 8 |  |  |
| Psychologist/Therapist | 9 |  |  |

**Meeting Records**

| **Meeting Type (1-3)** | **Date** | **Duration**  **(min)** | **Staff Occupation (code)** |
| --- | --- | --- | --- |
| **Initial Assessment (1)**  **Case Review:(2)**  **One-to-one meeting (3)** |  | **Initial assessment: Enter 60.**  **Case Review:**  **Enter 60.**  **One-to-one: enter exact time** |  |
|  |  |  |  |
|  |  |  |  |
|  |  |  |  |
|  |  |  |  |
|  |  |  |  |
|  |  |  |  |
|  |  |  |  |
|  |  |  |  |
|  |  |  |  |

**Scheduled Conversations**

(Planned conversations to monitor the person on ACCT, and observations during ACCT period)

We will calculate the total time each participant spends having these types of contact.

Please list:

- Frequency of contact (e.g. every 30 min)
- Average duration of the individual contacts (e.g. spends 10 minutes with the participant)
- Start date and end date
- Staff Occupation

Where can I find this information? ACCT folders

| **Contact Type** | **Frequency (min)** | **Duration (min)** | **Start date** | **End Date** | **Staff Occupation (code)** |
| --- | --- | --- | --- | --- | --- |
| **Scheduled conversations (1)/**  **Observation (2)** |  |  |  |  |  |
|  |  |  |  |  |  |
|  |  |  |  |  |  |
|  |  |  |  |  |  |
|  |  |  |  |  |  |
|  |  |  |  |  |  |
|  |  |  |  |  |  |
|  |  |  |  |  |  |
|  |  |  |  |  |  |
|  |  |  |  |  |  |

**2. Self-Harm Support Items**

Record all non-clothing items given to support self-harm prevention.

For example: distraction pack, stress balls, books

Where can I find this information? ACCT folder, SystmOne

Keep descriptions consistent (e.g. always write ‘distraction pack’)

| **Date** | **Item Description** |
| --- | --- |
|  |  |
|  |  |
|  |  |
|  |  |
|  |  |
|  |  |
|  |  |
|  |  |
|  |  |
|  |  |
|  |  |

**3. Alternative clothing Provided**

Where can I find this information?

ACCT folder/Safer Custody log/NOMIS

| **Date** | **Clothing Type** |
| --- | --- |
|  |  |
|  |  |
|  |  |
|  |  |
|  |  |

**4. Professional One-to-One Healthcare Contact Inside Prison**

Providers may be based inside the prison or may come into the prison to deliver a service.

Record number of contacts with each professional over study period and average duration of contacts

One to one therapy appointments are recorded separately on the concomitant therapy log.

If contact with other healthcare specialists add details to end of form

Where can I find this information? SystmOne

| **Professional** | **No. of Contacts** | **Avg duration (min)** |
| --- | --- | --- |
| GP/prison doctor |  | 10 |
| Practice Nurse/prison nurse/nurse practitioner**/**primary care nurse |  | 10 |
| Psychiatrist |  | 30 |
| Psychiatric Nurse |  | 30 |
| Health Care Assistant |  | 10 |
| Occupational Therapist |  |  |
| Drug and alcohol treatment Staff/CARAT staff **(**Counselling, Assessment, Referral, Advice and Throughcare) |  |  |
| Dentist |  | 20 |
| Optician |  | 20 |
| Chiropodist |  |  |
| Physiotherapist |  | 30 |
| Chaplain |  |  |
| Midwife |  | 30 |
| Podiatrist |  | 30 |
| Smoking cessation facilitator |  |  |
| Radiographer |  | 30 |
| Doctor for GUM clinic (sexual health) |  | 30 |
| Nurse for GUM clinic (sexual health) |  | 30 |
| Doctor for pain clinic |  | 30 |
| Nurse for pain clinic |  | 30 |
| Other: |  |  |
| Other: |  |  |
| Other: |  |  |
| Other: |  |  |
| Other: |  |  |

**5. Physical treatment for Self-Harm:**

**List resources used during professional treatment**

(E.g. cleaning liquids/wipes, dressings, steri-strips, stitches)

Where can I find this information? SystmOne

| **Date of Appointment** | **Item** | **Quantity** |
| --- | --- | --- |
|  |  |  |
|  |  |  |
|  |  |  |
|  |  |  |
|  |  |  |
|  |  |  |
|  |  |  |
|  |  |  |
|  |  |  |
|  |  |  |

**6. Healthcare Admission Inside Prison**

This concerns transfer to the healthcare wing inside the prison**.**

Where can I find this information? Check with the individual prisons

| **Reason for stay** | **Start Date** | **End Date** |
| --- | --- | --- |
|  |  |  |
|  |  |  |
|  |  |  |
|  |  |  |
|  |  |  |
|  |  |  |

**7. Hospital Admissions (Outside Prison, Overnight)**

**Please Note:**

- Reason for stay (including medical speciality)
- Date the participant entered and left hospital
- Number of escorts that were needed to accompany the prisoner/patient when they left AND returned to prison.

Create separate lines for each different hospital stay.

This section includes elective hospital stays (scheduled in advance) and emergency stays.

‘Bed watches’ is prison used to describe when prison staff need to escort and stay with people who are in hospital.

Where can I find this information? SystmOne

|  |  |  |  |  | **BED WATCH** | |
| --- | --- | --- | --- | --- | --- | --- |
| **Admission Type (1/2)** | **Reason** | **Start Date** | **End Date** | **No. of Escorts** | **Staff Grade** | **Staff Time** |
| Elective (1)/  Emergency (2) |  |  |  | Minimum standard escort is 2 officers there and back (4 in total). If individual information unavailable, enter 4 |  |  |
|  |  |  |  |  |  |  |
|  |  |  |  |  |  |  |
|  |  |  |  |  |  |  |

**8. Outpatient/A&E Attendance (Outside Prison)**

Where can I find this information? SystmOne

| **Type (1/2)**  Outpatient (1)/  A&E (2) | **Reason for Visit**  Include appointment type, with whom, and what appointment included e.g. X-ray | **Duration (min)** | **Escort Number (to & from)**  Enter 4 if specific number is not recorded |
| --- | --- | --- | --- |
|  |  |  |  |
|  |  |  |  |
|  |  |  |  |
|  |  |  |  |
|  |  |  |  |

**9. Segregation**

Record**:**

- Any time spent on segregation
- Whether under Rule 45 segregation order (i.e. in segregation for own protection).

All persons in segregation should also be visited daily by healthcare and a senior manager.

Where can I find this information?

This will vary by prison. It may include NOMIS

| **Start Date** | **End Date** | **Rule 45? (Yes/No)** | **Healthcare Visit Time (total min)** | **Senior Manager Visit Time (total min)** |
| --- | --- | --- | --- | --- |
|  |  |  |  |  |
|  |  |  |  |  |
|  |  |  |  |  |
|  |  |  |  |  |
|  |  |  |  |  |
|  |  |  |  |  |

**For Resource Use Calculation, Also Refer To:**

- Concomitant therapy record
- Concomitant medications record

*This tool was developed by Greater Manchester NHS Mental Health Trust and the University of Manchester*
